# Perinatal outcomes among multiracial women compared to both Monoracial Majority and Minority groups in a Medicaid sample: 2017–2019

**DOI:** 10.21203/rs.3.rs-6835988/v1

**Published:** 2025-08-03

**Authors:** Karen M. Tabb, Andrea Pangori, Anca Tilea, Gloria Sugg, Stephanie V. Hall, Hsiang Huang, Vanessa Dalton, Kara Zivin

**Affiliations:** University of Illinois at Urbana-Champaign; University of Michigan; University of Michigan; University of Illinois at Urbana-Champaign; University of Michigan; Harvard Medical School, Cambridge Health Alliance; University of Michigan; University of Michigan

## Abstract

**Objectives:**

This study aims to explore prevalence of perinatal mental health conditions among Multiracial women in the United States compared to Monoracial Minority and Monoracial Majority women.

**Methods:**

We analyzed Medicaid claims data from 2016–2020 for 1,863,150 delivering women across 30 states, focusing on those with continuous enrollment from nine months prior to delivery to three months postpartum. We categorized women as Multiracial (two or more races), Monoracial Majority, Monoracial Minority, and Hispanic.

**Results:**

Multiracial women compared to Monoracial Minority women demonstrated higher rates of mental health conditions. Multiracial women were 16% less likely than Monoracial Majority (OR: 0.84, 95% CI: 0.79–0.89) and 45% more likely than Monoracial Minority (OR: 1.45, 95% CI: 1.34–1.56) to have a perinatal depression diagnosis. Multiracial women had higher suicidal ideation diagnoses compared to both Monoracial Minority and Monoracial Majority women.

**Conclusions:**

The findings highlight disparities in perinatal mental health conditions among Multiracial female Medicaid beneficiaries.

**Policy Implications:**

Researchers and policymakers should work further to address the unique mental health needs of perinatal Multiracial women, with an emphasis on addressing suicidal ideation.

## Introduction

Perinatal mental health conditions increased dramatically in the United States (U.S.) in recent years.^1^ Significant disparities exist in perinatal mental health conditions complications such as adverse birth outcomes.^2^ Additionally, racial/ethnic groups have differential access to mental health care.^3,4^ Multiracial women represent the fastest growing demographic group in the U.S, yet despite documented perinatal mental health disparities by race/ethnicity, few studies examine Multiracial populations using either surveys or claims data. Recent investigations found increased perinatal mental health conditions for Multiracial women compared to White women.^5–7^ The present study analyzes Medicaid claims data to document prevalence of perinatal mental health conditions among Multiracial women compared to single race non-White minority (Monoracial Minority) and single race White majority (Monoracial Majority) women.

## Methods

This cross-sectional study uses national Medicaid administrative claims from April 2016 through March 2020 to assess outcomes related to deliveries that occurred between January 2017 and December 2019. We conducted the study in accordance with the Declaration of Helsinki. The University of Michigan Institutional Review Board approved the study under HUM00204182. We restricted our analysis to women with 9 months of continuous enrollment prior and 3 months after delivery. Medicaid’s Data Quality Atlas assesses race/ethnicity data quality collected in each state.^8^ We included states with “low” and “medium” concern race/ethnicity quality, leaving 32 states in 2017 and 30 states in 2018 and 2019.

Our primary covariate of interest is race/ethnicity, found in the demographic beneficiary table and categorized as American Indian and Alaskan Native (AIAN) non-Hispanic, Asian non-Hispanic, Black non-Hispanic, Hawaiian/Pacific Islander non-Hispanic, Hispanic (all races), Multiracial non-Hispanic, Other non-Hispanic, or White non-Hispanic. The Research Data Assistance Center (ResDAC) assigns these categories using an established algorithm.^9^

We found more than one record for race/ethnicity for less than 1% of eligible beneficiaries, within and across years and states. To consolidate race/ethnicity information within a beneficiary’s record, we implemented a revised algorithm to categorize race/ethnicity. If an individual had more than one race/ethnicity record within a year and one of the records was Hispanic, we classified the individual as Hispanic, following ResDAC’s algorithm. If we found at least two different not Hispanic records within the year, we classified the individual as Multiracial.

Once we applied the algorithm to all years individually, we applied the same algorithm across years. The algorithm assigned one race/ethnicity record to each beneficiary. We categorized these races/ethnicities into 4 groups: 1) Monoracial Majority (White non-Hispanic), 2) Monoracial Minority (Black, Asian, AIAN, Hawaiian/Pacific Islander, and Other, non-Hispanic), 3) Multiracial, and 4) Hispanic.^10^

Our outcomes of interest include perinatal mood and anxiety disorder (PMAD), anxiety, depression, suicidal ideation, post-traumatic stress disorder (PTSD), and bipolar disorder. We identified these outcomes using one inpatient or two outpatient claims in the 9 months prior or 3 months after the delivery using International Classification of Diseases, Tenth Revision, Clinical Modification codes. Our demographic variables include age and Bateman Obstetric Comorbidity Index (OBCMI). OBCMI is a validated risk score for severe maternal morbidity (SMM) with higher score indicating increased SMM risk.^11^

### Statistical analysis

We first summarized demographic characteristics overall and by race/ethnicity groups for all individuals. We applied unadjusted logistic regression models to quantify race/ethnicity differences for each outcome. We first used the Monoracial Majority group as the reference group, then repeated these models with Monoracial Minority as the reference group applying a Bonferroni correction to adjust for multiple comparisons. We used two-sided statistical tests with an alpha level of 0.05 for all statistical analyses. We conducted data management in SAS version 9.4 (SAS Institute) and statistical analyses in R version 4.3.2.

## Results

From 2017 to 2019, we identified 1,863,150 delivering women, including 714,534 (38.4%) Monoracial Majority, 628,357 (33.7%) Monoracial Minority, 478,254 (25.7%) Hispanic, 12,193 (0.6%) Multiracial, and 29,812 (1.6%) with missing race/ethnicity value. Since we did not include unknown race in the models, our analytical sample consisted of 1,833,338 women. Supplemental Table 1 describes age and OBCMI. Multiracial and Hispanic groups were younger than Monoracial Majority and Minority, but OBCMI score remainedsimilar across race/ethnicity groups.

**Figure 1:**
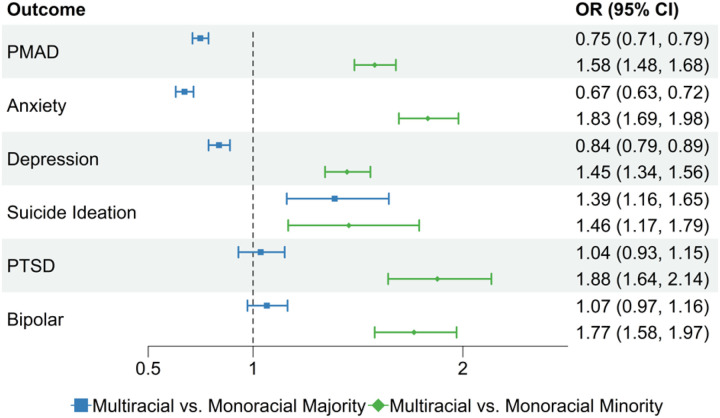
Forest Plot of perinatal mental health conditions among Multiracial Medicaid beneficiaries *Footnote: Confidence intervals that cross the dotted line at 1 did not reach statistical significance at the 0.05 alpha level.

As shown in Fig. 1, we found that Multiracial women were 25% less likely than Monoracial Majority (OR: 0.75, 95% CI: 0.71–0.79) and 58% more likely than Monoracial Minority (OR: 1.58, 95% CI: 1.48–1.68) to have a PMAD diagnosis. Multiracial women were 33% less likely than Monoracial Majority (OR: 0.67, 95% CI: 0.63–0.72) and 83% more likely than Monoracial Minority (OR: 1.83, 95% CI: 1.69–1.98) to have a perinatal anxiety diagnosis. Multiracial women were 16% less likely than Monoracial Majority (OR: 0.84, 95% CI: 0.79–0.89) and 45% more likely than Monoracial Minority (OR: 1.45, 95% CI: 1.34–1.56) to have a perinatal depression diagnosis.

We also found that Multiracial women were 39% more likely than Monoracial Majority (OR: 1.39, 95% CI: 1.16–1.65) and 46% more likely than Monoracial Minority (OR: 1.46, 95% CI: 1.17–1.79) to have suicidal ideation. We found no significant difference in likelihood of perinatal PTSD diagnosis between Multiracial and Monoracial Majority women (OR: 1.04, 95% CI: 0.93–1.15) and found that Multiracial individuals were 88% more likely to have a perinatal PTSD diagnosis than Monoracial Minority (OR: 1.88, 95% CI: 1.64–2.14). Similarly, we found no significant difference in likelihood of a perinatal bipolar diagnosis between Multiracial and Monoracial Majority women (OR: 1.07, 95% CI: 0.97–1.16) and found that Multiracial women were 77% more likely to have a perinatal bipolar disorder diagnosis than Monoracial Minority (OR: 1.77, 95% CI: 1.58–1.97).

## Discussion

This study examined perinatal mental health conditions among Multiracial women compared to single race/ethnicity peers. Our findings align with recent studies reporting increased burden of these conditions among Multiracial women.^5–7^ As a major literature contribution, we compared Multiracial and Monoracial Medicaid beneficiaries and found that diagnoses for suicidal ideation were significantly greater for Multiracial women compared to White and non-White counterparts. This suicidal ideation finding is alarming and needs urgent replication.

Among Multiracial women enrolled in Medicaid with PTSD and bipolar disorder diagnoses, we found no differences from Monoracial populations, contrasting with a recent study highlighting an increased burden of bipolar disorder diagnosis among Multiracial women in California.^12^ In our Medicaid sample, Multiracial women experienced greater diagnoses for PMAD, anxiety, and depression than the overall sample but less than Monoracial Majority women.

A limitation of this study is that our analytical sample may represent those with greater health care access, as we restricted Medicaid claims data to beneficiaries who gave birth with continuous enrollment nine months prior to delivery and three months post-delivery. These data indicate clinical diagnosis, not prevalence of disease, and therefore may not account for disparities in detection due to differential access to care or other biases within the health care system. Privately insured or uninsured women may have different results.

Given past documented associations between Multiracial individuals and poor perinatal mental health outcomes,^5^ future work should aim to determine clinical practices and public health education campaigns.

## Conclusions

Since little information existed about the rate of depression and suicidal ideation diagnoses and its determinants among Multiracial women compared to Monoracial Minority and Monoracial Majority peers prior to this study, we began to fill the knowledge gap of Multiracial health disparities. Results from this study represent the start of a scientific journey to explore potential risks and unique needs among perinatal Multiracial women.

## Supplementary Material

Supplementary Files

This is a list of supplementary files associated with this preprint. Click to download.

- SupplementalTable1.docx

## Data Availability

The data that support the findings of this study are available from the Centers for Medicare & Medicaid Services. Restrictions apply to the availability of these data, which were used under DUA RSCH-2022–57879 for this study, and thus are not publicly available.
